# Access to care in Afghanistan after august 2021: a cross-sectional study exploring Afghans’ perspectives in 10 provinces

**DOI:** 10.1186/s13031-024-00594-5

**Published:** 2024-04-22

**Authors:** Martina Valente, Alessandro Lamberti-Castronuovo, Francesca Bocchini, Yasir Shafiq, Monica Trentin, Michela Paschetto, Ghulam Ali Bahdori, Jan Agha Khadem, Mirza Sayed Nadeem, Mohammand Hanif Patmal, Mohammad Tawoos Alizai, Francesco Barone-Adesi, Rossella Miccio, Luca Ragazzoni

**Affiliations:** 1 CRIMEDIM - Center for Research and Training in Disaster Medicine, Humanitarian Aid and Global Health, Università del Piemonte Orientale, via Bernardino Lanino, 1, Novara, 28100 Italy; 2 Department for Sustainable Development and Ecological Transition, Università del Piemonte Orientale, via Duomo, 6, Vercelli, 13100 Italy; 3 EMERGENCY ONG ONLUS, via Santa Croce, 19, Milan, 20122 Italy; 4 Department of Translational Medicine, Università del Piemonte Orientale, via Solaroli, 17, Novara, 28100 Italy; 5 Department of Community Health Sciences and Centre of Excellence for Trauma and Emergencies, Aga Khan University, Karachi, Pakistan; 6 EMERGENCY ONG ONLUS, Anabah Hospital, Anabah, Afghanistan; 7 EMERGENCY ONG ONLUS, Kabul Hospital, Kabul, Afghanistan; 8 EMERGENCY ONG ONLUS, Lashkar-Gah Hospital, Lashkar-Gah, Afghanistan

**Keywords:** Afghanistan, Questionnaire, Universal coverage, Conflict, Humanitarian

## Abstract

**Background:**

The Taliban takeover in August 2021 ended a decades-long conflict in Afghanistan. Yet, along with improved security, there have been collateral changes, such as the exacerbation of the economic crisis and brain drain. Although these changes have altered the lives of Afghans in many ways, it is unclear whether they have affected access to care. This study aimed to analyse Afghans’ access to care and how this access has changed after August 2021.

**Methods:**

The study relied on the collaboration with the non-governmental organisation EMERGENCY, running a network of three hospitals and 41 First Aid Posts in 10 Afghan provinces. A 67-item questionnaire about access to care changes after August 2021 was developed and disseminated at EMERGENCY facilities. Ordinal logistic regression was used to evaluate whether access to care changes were associated with participants’ characteristics.

**Results:**

In total, 1807 valid responses were returned. Most respondents (54.34%) reported improved security when visiting healthcare facilities, while the ability to reach facilities has remained stable for the majority of them (50.28%). Care is less affordable for the majority of respondents (45.82%). Female respondents, those who are unmarried and not engaged, and patients in the Panjshir province were less likely to perceive improvements in access to care.

**Conclusions:**

Findings outline which dimensions of access to care need resource allocation. The inability to pay for care is the most relevant barrier to access care after August 2021 and must therefore be prioritised. Women and people from the Panjshir province may require ad hoc interventions to improve their access to care.

**Supplementary Information:**

The online version contains supplementary material available at 10.1186/s13031-024-00594-5.

## Background

Afghanistan’s health system has been severely impacted by more than four decades of war. For years, much of the country’s resources have been directed to defence and military operations rather than to improving healthcare [1] to the extent that Non-Governmental Organisations (NGOs) have provided more health services than the state [2]. Political unrest, along with frequent disasters and epidemics, including the COVID-19 pandemic, has severely impaired health service delivery and population’s access to basic care [3]. Nevertheless, significant progress has been made over the last two decades: maternal mortality has declined from approximately 1,450 deaths per 100,000 live births in 2000 to 638 deaths per 100,000 live births in 2017 [4, 5], under-five mortality rate has decreased from 181 deaths per 1000 live births in 1990 to 91 deaths per 1000 live births in 2015 [6], and life expectancy has increased [7] thanks to far-reaching reforms of the country’s health services [8, 9]. By the beginning of 2021, Afghanistan was on its way to expand the scope of services through an integrated reform that would have moved the country towards Universal Health Coverage (UHC) [10].

The situation abruptly changed after the Taliban takeover in August 2021. Humanitarian funding was cut overnight, Afghan international assets were frozen in banks and many organisations along with national and international healthcare professionals left the country. This set back the gains made over the last years [10], forcing people further into poverty and worsening an already under-resourced public health system. Access to basic rights, including healthcare and education, is still a major concern in the country, particularly for some vulnerable populations. After August 2021, ethnic minorities have increasingly been the target of acts of violence and faced discrimination in accessing basic care, especially in the Panjshir province, where episodes of fighting are still being recorded [11]. Women were banned from working at NGOs and from attending university, making Afghanistan the only country in the world where women are denied education [12], a fact that is particularly troubling given that investing in education can reduce poverty and improve public health, healthcare access, and economic growth [1, 13].

Along with the negative impacts, the political transition in August 2021 has led to the cessation of a 40-years long conflict and the reopening of areas long used as battlefields. This allows humanitarian health workers and researchers to access previously inaccessible communities, and people to travel roads more safely. The withdrawal of United States (US) and North Atlantic Treaty Organisation (NATO) troops in Afghanistan after nearly 20 years resulted in a renewed sense of peace, though it did not translate into economic growth [14]. If the end of the war and the socio-political changes in the country have undoubtedly left a deep mark on the Afghan population, it is still unknown whether such changes have affected access to care. Much of the health-related scientific literature focusing on Afghanistan published after August 2021 consists of clinical highly localised studies, or opinion pieces and commentaries. Studies examining access to care of Afghans predominantly refer to the refugee population in foreign countries and no study to date has explored changes in access to care after August 2021.

The aim of this study was to analyse Afghans’ access to care and how this access has changed under the new socio-political developments. The results will shed light on healthcare challenges faced by the Afghan population and can pave the way for devising health reforms that will have a marked and direct positive impact on Afghan communities.

## Methods

### Study design and timeframe

This cross-sectional study was conducted from June 2022 to March 2023 in the context of a broader collaboration between CRIMEDIM, Center for Research and Training in Disaster Medicine, Humanitarian Aid and Global Health, and EMERGENCY ONG (EMERGENCY). All phases of the project were jointly carried out by collaborators from both institutions, in close cooperation with EMERGENCY medical staff and field officers in Afghanistan. Data collection took place in September and October 2022, and relied on the extensive network of EMERGENCY facilities and staff in Afghanistan. The study protocol was approved by the Afghanistan Institutional Review Board (IRB Code No: A.0823.419).

### Study setting

The geographical outreach of the study corresponds to the areas where EMERGENCY is located in the country, namely the provinces of Ghazni, Helmand, Kabul, Kapisa, Lagman, Logar, Paktia, Panjshir, Parwan and Wardak^1^.

**Fig. 1:**
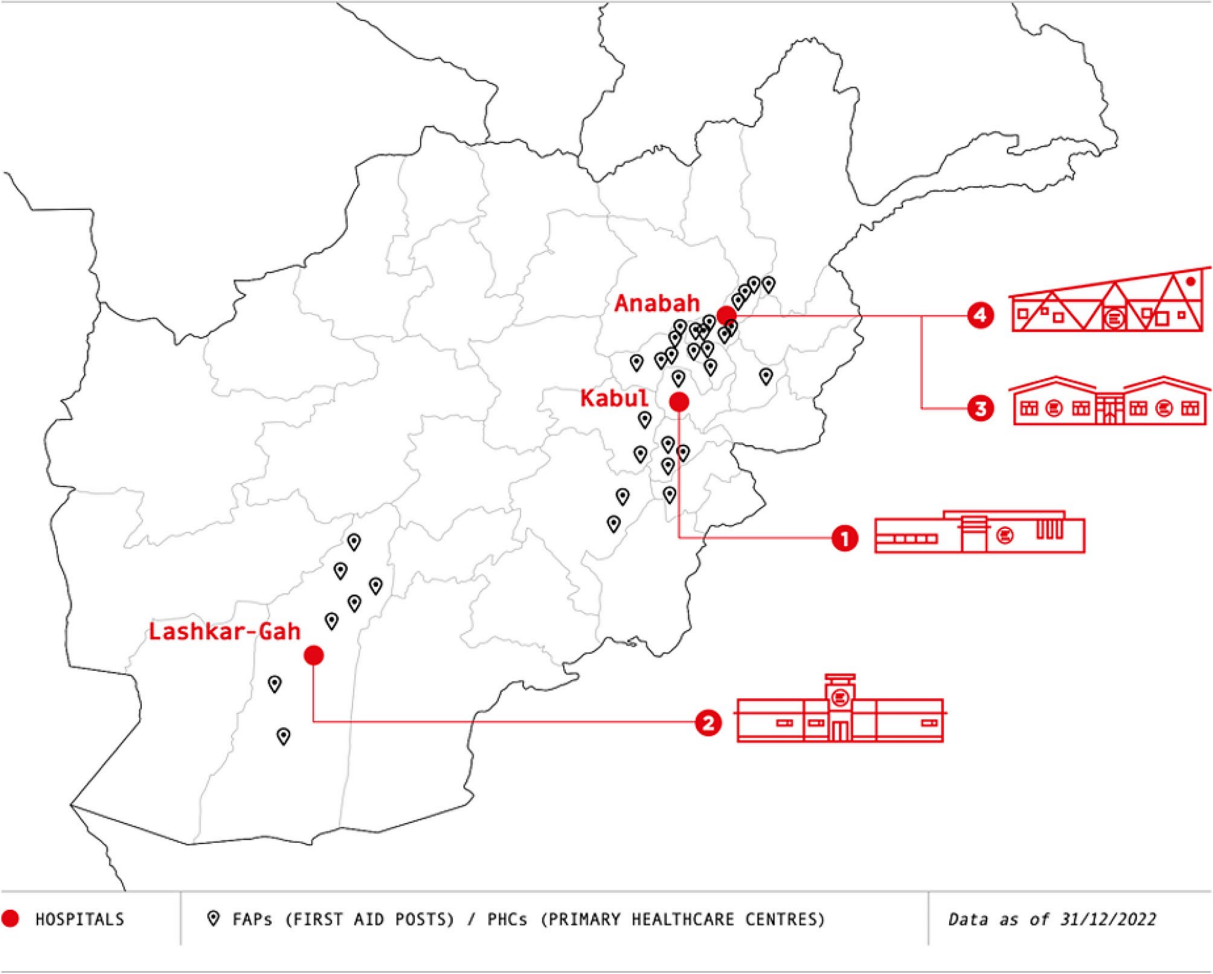
Map showing the distribution of EMERGENCY facilities in Afghanistan Figure 1 reproduced with permission from EMERGENCY

EMERGENCY started its activity in Afghanistan in 1999 and has become today a key health reference point for the population, alongside the public health system. It counts three main centres in the country: surgical centre for war victims in Kabul (Kabul province), surgical centre for war victims in Lashkar-Gah (Helmand province), and both a surgical centre and a paediatric and maternity centre in Anabah (Panjshir province). Besides the three main centres, EMERGENCY has a network of 41 First Aid Posts (FAPs) and Primary Healthcare Centres (PHCs) across 11 provinces, each falling in the catchment area of one of the three main centres in the Panjshir, Kabul and Helmand provinces (Fig. 1).

Although the war affected all areas of Afghanistan, the Panjshir province had not been particularly affected by active fighting over the past 20 years. After the Taliban takeover, episodes of fighting have increased in the Panjshir province due to resistance undertaken by the opposition. Conversely, the Kabul and Helmand provinces have witnessed a sharp decline in combat activity as compared to the past.

### Data collection tools

Information was collected via a 67-item questionnaire (Supplementary File 1). The questionnaire was informed by the theoretical framework from Levesque et al. (2013) [15] and aimed to explore access to care trends over the past years, and if and how these trends have modified after the change of government in August 2021. The questionnaire was made up of four main sections: the first enquired about information on the facility and location of data collection, the second collected demographic information on the participant, the third assessed access to care in general, and the fourth examined obstacles and changes in access to care. The questionnaire encompassed multiple-choice and ranking questions; it was developed in English and translated into Dari and Pashto through a double translation process. Before administering it, it was piloted by a pool of scholars and local healthcare workers. It took participants about 20 min to complete the questionnaire. To facilitate data collection, the English, Dari, and Pashto versions of the questionnaire were transferred to a digital smartphone platform (i.e., KoboCollect).

### Study population and sampling

Participants were recruited at EMERGENCY facilities (i.e., hospitals, FAPs and PHCs) in ten provinces of Afghanistan (i.e., Ghazni, Helmand, Kabul, Kapisa, Lagman, Logar, Paktia, Panjshir, Parwan and Wardak). Sample size calculation was performed considering the 2022 monthly average number of visits in each facility (for hospitals, outpatients visits were considered). The final sample size for both FAPs/PHCs and hospitals was adjusted for 25% non-response rate. Sample size estimates per each facility were obtained, for a total sample size of 1837 participants (551 for the Kabul catchment area, 687 for the Anabah catchment area, and 599 for the Lashkar-Gah catchment area). Sample size calculation was performed to obtain a sample that was representative of EMERGENCY’s patients across catchment areas, and to plan the recruitment of data collectors per each facility. To participate in the study, the following inclusion criteria applied: being 18 years old or older, having lived in Afghanistan for the past three years, being in EMERGENCY premises at the point of data collection, speaking either Dari, Pashto or English, and providing informed consent.

### Data collection

According to each facility’s estimated sample size, one or more data collectors per facility were recruited from EMERGENCY staff in Afghanistan. Data collectors attended a one-day training session on the significance and objective of the study, ethical principles, and on how to use KoboCollect. The completion of the questionnaire was on a voluntary basis and data collectors disseminated the questionnaire to patients visiting their facility upon their consent and willingness to participate, as well as to any interested accompanying persons; data collectors dedicated one or more days per week to the collection of data (at their discretion and depending on time availability). Patients were offered the survey if they met the eligibility criteria and if their health status allowed it (e.g., the questionnaire was not administered to those who presented with open wounds, urgent conditions requiring immediate referral or hospitalisation, or those in pain). Data was collected from mid-September to the end of October 2022. The research team closely monitored data collection and was available throughout the whole process. All responses collected across different provinces were automatically transferred in electronic format to the research team’s Kobo account. Verbal consent was obtained by participants prior to starting data collection. All pertinent ethical considerations were explained to participants at the beginning of data collection. No personal identifiers were collected from participants at any point during the study.

### Data analysis

#### Variables

Four dependent variables were considered to explore changes in access to care after August 2021: (i) change in the sense of security when visiting health facilities; (ii) change in the ability to seek care; (iii) change in the ability to reach health facilities; (iv) change in the ability to pay for care, intended as the overall ability to afford services and medicines. Responses were coded as “0” when they referred to a perceived worsening of the situation, “1” when they referred to neither a worsening nor an improvement, and “2” when they referred to a perceived improvement in the situation. For each dependent variable, we evaluated the association with the following independent variables: (i) geographical area, categorised as per the catchment area of the three main centres (Anabah, Kabul, Lashkar-Gah); (ii) gender (female, male); (iii) marital status (married, engaged, not married or engaged, widow); (iv) location (rural, urban); (v) education (formal, informal or no education); (vi) financial difficulties for spending on healthcare (yes, no); and (vii) age (being younger than 34 years old, being 35–54 years old, and being older than 55 years old).

### Statistical analysis

Questionnaire responses that were incomplete for more than 80% and invalid entries (e.g., responses from participants younger than 18 years old) were excluded from the analysis. Descriptive statistics were used to summarise demographic information and estimate the main changes in access to care after August 2021. Ordinal logistic regression analysis was performed to evaluate whether access to care after August 2021 was associated with some characteristics of the participants. Responses with missing values for the dependent variables were excluded from the regression analysis. Both univariable and multivariable analyses were performed, obtaining Odds Ratios (ORs) and 95% Confidence Intervals (CIs). Data analysis was performed using Stata (version 17.0).

## Results

### Demographics

A total of 1832 responses were obtained from patients or people accompanying them at EMERGENCY facilities (response rate of 99.73%). After the exclusion of 25 invalid responses, the final sample size was 1807. The majority of the participants were male (71.7%; *n* = 1295) and slightly over half were younger than 34 years old (52.3%; *n* = 849). Most of the participants lived in rural areas (82.9%, *n* = 1498) and were married (81.9%, *n* = 1469). When it comes to the reasons for visiting an EMERGENCY facility, respondents could select more than one option and reported being there for medical examination (32.0%, *n* = 702), drugs prescription (19.9%, *n* = 359), urgent care (23.9, *n* = 431), health system navigation (i.e., help to orient themselves in the health system) (3.4%, *n* = 61), general help (3.4%, *n* = 61) or to accompany someone (24.9%, *n* = 450) (Table 1).

**Table 1:** Demographic information on the study participants

Demographic variables	*N*(%)
**Gender** (*N* = 1807)	
Female	512 (28.3)
Male	1295 (71.7)
**Age** (*N* = 1624)	
< 34	849 (52.3)
35–54	626 (38.5)
> 55	149 (9.2)
Geographical catchment area (*N* = 1792*)	
Anabah	702 (39.2)
Kabul	498 (27.8)
Lashkar-Gah	592 (33.0)
**Marital status** (*N* = 1793)	
Married	1469 (81.9)
Engaged	117 (6.5)
Not married or engaged	156 (8.7)
Widow	51 (2.8)
**Head of household** (*N* = 1806)	
Yes	1055 (58.4)
No	750 (41.6)
**Education** (*N* = 1791)	
Formal education	1219 (68.1)
Informal or no education	572 (31.9)
**Residence area** (*N* = 1807)	
Urban	299 (16.6)
Rural	1498 (82.9)
Unknown	10 (0.6)
**Employment** (*N* = 1799)	
Employer	32 (1.8)
Self-employed	359 (20.0)
Salaried worker, private sector	99 (5.5)
Salaried worker, public sector	128 (7.1)
Subsistence farmer	529 (29.4)
Unpaid family worker	363 (20.2)
Day laborer	289 (16.1)
Reason to visit the EMERGENCY health facility** (*N* = 1804)	
Medical examination	702 (32)
Drugs prescription	359 (19.9)
Health system navigation	61 (3.4)
Urgent care	431 (23.9)
Accompanying someone	450 (24.9)
General help	61 (3.4)
Other	106 (5.88)

*Total entries for the regression analysis

**More than one option was possible

### Perceived change in access to care

The majority of respondents (54.34%, *n* = 976) reported that their sense of security when accessing care improved after August 2021, especially in the Lashkar-Gah catchment area (92.4%, *n* = 547) and for males (66.56%, *n* = 862). When considering changes in the ability to seek care, some respondents reported that it improved (35.63%, *n* = 640), some reported that it worsened (33.63%, *n* = 604), and some others that it neither improved nor worsened (30.73%, *n* = 552). When it comes to changes in the ability to reach health facilities after August 2021, respondents largely reported that it neither improved or worsened (50.28%, *n* = 903), with the majority of patients from the Lashkar-Gah catchment area indicating an improvement (76.56%, *n* = 454). With the exception of respondents from the Lashkar-Gah area, study participants largely reported a worsening in the affordability of care. Ability to pay particularly affected respondents in the Anabah catchment area (73.83%, *n* = 522), and females (64.45%, *n* = 330) (Supplementary File 2).

### Regression analysis

#### Predictors of improved sense of security

The variable geographical area represents the different locations where the questionnaire was administered, with the Anabah catchment area as the reference category. Results show that individuals living in the Kabul and Lashkar-Gah areas are more likely to report an improvement in their sense of security than to those living in Anabah (OR: 5.97, 95%CI: 4.53–7.86; OR: 30.40, 95%CI: 19.89–46.47). The results also show that males are more likely to report an improvement in their sense of security compared to females (OR: 1.72, 95%CI: 1.32–2.24), that those who are not married nor engaged are more likely to report an improvement in their sense of security compared to those who are married (OR: 1.54, 95%CI: 1.02–2.32), and that those who are widow are less likely to perceive an improvement in their sense of security compared to those who are married (OR: 0.52, 95%CI: 0.28–0.95) (Table 2).

**Table 2:** Association between improved sense of security and baseline characteristics of participants after August 2021. The dependent variable is the perceived change in the sense of security: worsened (0), neither worsened nor improved (1), improved (2).

Variables	Univariable		Multivariable	
	OR (95%CI)	*p*-value	OR (95%CI)	*p*-value
**Geographical catchment area**				
Anabah	1 (ref.)		1 (ref.)	
Kabul	7.35 (5.72–9.44)	0.000	5.97 (4.53–7.86)	0.000
Lashkar-Gah	56.75 (39.81–80.88)	0.000	30.40 (19.89–46.47)	0.000
**Gender**				
Female	1 (ref.)		1 (ref.)	
Male	6.12 (4.96–7.56)	0.000	1.72 (1.32–2.24)	0.000
**Marital status**				
Being married	1 (ref.)		1 (ref.)	
Being engaged	3.61 (2.26–5.74)	0.000	1.34 (0.74–2.43)	0.333
Not married or engaged	1.20 (0.88–1.65)	0.253	1.54 (1.02–2.32)	0.040
Widow	0.26 (0.16–0.43)	0.000	0.52 (0.28–0.95)	0.032
**Location**				
Rural	1 (ref.)		1 (ref.)	
Urban	1.09 (0.85–1.39)	0.491	0.79 (0.59–1.07)	0.129
**Education**				
Formal education	1 (ref.)		1 (ref.)	
Informal or no education	2.73 (2.22–3.37)	0.000	1.27 (0.97–1.65)	0.082
**Financial difficulties**				
Yes	1 (ref.)		1 (ref.)	
No	1.28 (0.96–1.70)	0.089	0.99 (0.70–1.40)	0.939
**Age**				
< 34 years	1 (ref.)		1 (ref.)	
35–54 years	0.69 (0.57–0.84)	0.000	0.83 (0.65–1.06)	0.137
> 55 years	0.76 (0.55–1.05)	0.094	0.68 (0.45–1.02)	0.062

#### Predictors of improved ability to seek care

As for the ability to seek care, individuals in the areas of Kabul and Lashkar-Gah are more likely to report an improvement in their ability to seek care compared to those in Anabah (OR: 2.32, 95%CI: 1.80–2.98; OR: 37.19, 95%CI: 25.56–54.13). The results also show that those who are not married nor engaged are more likely to report an improvement in their ability to seek care compared to those who are married (OR: 1.60, 95%CI: 1.10–2.35) and that those who are widow are less likely to report an improvement in their ability to seek care compared to those who are married (OR: 0.40, 95%CI: 0.20–0.78). Furthermore, those who have never encountered financial difficulties in paying for care are more likely to report an improvement in their ability to seek care compared to those who have encountered financial difficulties in paying for care (OR: 2.03, 95%CI: 1.48–2.78) (Table 3).

**Table 3:** Association between improved ability to seek care and baseline characteristics of participants after August 2021. The dependent variable is the perceived change in the ability to seek care: worsened (0), neither worsened nor improved (1), improved (2)

Variable	Univariable		Multivariable	
	OR (95%CI)	*p*-value	OR (95%CI)	*p*-value
**Geographical catchment area**				
Anabah	1 (ref.)		1 (ref.)	
Kabul	2.22 (1.78–2.77)	0.000	2.32 (1.80–2.98)	0.000
Lashkar-Gah	35.55 (25.53–44.10)	0.000	37.19 (25.56–54.13)	0.000
**Gender**				
Female	1 (ref.)		1 (ref.)	
Male	3.18 (2.62–3.86)	0.000	0.89 (0.69–1.15)	0.373
**Marital status**				
Being married	1 (ref.)		1 (ref.)	
Being engaged	4.06 (2.71–6.09)	0.000	1.21 (0.74–1.99)	0.444
Not married or engaged	1.46 (1.08–1.98)	0.014	1.60 (1.10–2.35)	0.014
Widow	0.25 (0.14–0.44)	0.000	0.40 (0.20–0.78)	0.008
**Location**				
Rural	1 (ref.)		1 (ref.)	
Urban	1.02 (0.81–1.29)	0.854	0.86 (0.65–1.13)	0.276
**Education**				
Formal education	1 (ref.)		1 (ref.)	
Informal or no education	2.30 (1.90–2.78)	0.000	0.92 (0.72–1.17)	0.502
**Financial difficulties**				
Yes	1 (ref.)		1 (ref.)	
No	2.36 (1.80–3.08)	0.000	2.03 (1.48–2.78)	0.000
**Age**				
< 34 years	1 (ref.)		1 (ref.)	
35–54 years	0.60 (0.49–0.72)	0.000	0.88 (0.70–1.11)	0.287
> 55 years	0.85 (0.62–1.17)	0.320	0.89 (0.60–1.32)	0.573

#### Predictors of improved ability to Reach Care

As for the ability to reach care, individuals in the areas of Kabul and Lashkar-Gah are more likely to report an improvement in their ability to reach health facilities compared to those in Anabah (OR: 2.41, 95%CI: 1.84–3.17; OR: 24.12, 95%CI: 16.65–34.95). The results also show that those who are not married but are engaged are more likely to report an improvement in their ability to reach health facilities compared to those who are married (OR: 1.08, 95%CI: 1.08–3.11) (Table 4).

**Table 4:** Association between improved ability to reach health facilities and baseline characteristics of participants after August 2021. The dependent variable is the perceived change in the ability to reach health facilities: worsened (0), neither worsened nor improved (1), improved (2)

Variable	Univariable		Multivariable	
	OR (95%CI)	*p*-value	OR (95%CI)	*p*-value
**Geographical catchment area**				
Anabah	1 (ref.)		1 (ref.)	
Kabul	2.53 (1.98–3.23)	0.000	2.41 (1.84–3.17)	0.000
Lashkar-Gah	22.58 (17.25–29.57)	0.000	24.12 (16.65–34.95)	0.000
**Gender**				
Female	1 (ref.)		1 (ref.)	
Male	3.47 (2.82–4.26)	0.000	1.11 (0.86–1.46)	0.410
**Marital status**				
Being married	1 (ref.)		1 (ref.)	
Being engaged	4.30 (2.86–6.46)	0.000	1.83 (1.08–3.11)	0.025
Not married or engaged	1.30 (0.96–1.78)	0.094	1.44 (0.98–2.14)	0.066
Widow	0.34 (0.21–0.57)	0.000	0.56 (0.31–1.01)	0.053
**Location**				
Rural	1 (ref.)		1 (ref.)	
Urban	1.09 (0.86–1.38)	0.487	0.91 (0.68–1.22)	0.537
**Education**				
Formal education	1 (ref.)		1 (ref.)	
Informal or no education	2.28 (1.88–2.78)	0.000	0.93 (0.72–1.20)	0.579
**Financial difficulties**				
Yes	1 (ref.)		1 (ref.)	
No	1.78 (1.35–2.35)	0.000	1.32 (0.94–1.84)	0.105
**Age**				
< 34	1 (ref.)		1 (ref.)	
35–54 years	0.59 (0.48–0.72)	0.000	0.86 (0.67–1.10)	0.210
> 55 years	0.88 (0.63–1.22)	0.455	0.90 (0.61–1.34)	0.610

#### Predictors of improved ability to pay for care

Lastly, the results show that individuals in the areas of Kabul and Lashkar-Gah are more likely to report an improvement in their ability to pay for care compared to those in Anabah (OR: 3.02, 95%CI: 2.29–3.98; OR: 46.65, 95%CI: 31.86–68.31). The results also show that those who are not married nor engaged are more likely to report an improvement in their ability to pay for care compared to those who are married (OR: 1.56, 95%CI: 1.05–2.31), that those living in urban areas are less likely to report an improvement in their ability to pay for care compared to those who live in rural areas (OR: 0.62, 95%CI: 0.46–0.83), and that those who have never encountered financial difficulties in paying for care are more likely to report an improvement in their ability to pay for care compared to those who have encountered financial difficulties in paying for care (OR: 2.31, 95%CI: 1.69–3.15) (Table 5).

**Table 5:** Association between improved ability to pay for healthcare and baseline characteristics of participants after August 2021. The dependent variable is the perceived change in the ability to pay for healthcare: worsened (0), neither worsened nor improved (1), improved (2)

Variable	Univariable		Multivariable	
	OR (95%CI)	*p*-value	OR (95%CI)	*p*-value
**Geographical catchment area**				
Anabah	1 (ref.)		1 (ref.)	
Kabul	2.71 (2.14–3.45)	0.000	3.02 (2.29–3.98)	0.000
Lashkar-Gah	36.30 (27.49–47.95)	0.000	46.65 (31.86–68.31)	0.000
**Gender**				
Female	1 (ref.)		1 (ref.)	
Male	3.37 (2.74–4.14)	0.000	0.86 (0.65–1.14)	0.293
**Marital status**				
Being married	1 (ref.)		1 (ref.)	
Being engaged	4.32 (2.96–6.29)	0.000	1.50 (0.94–2.40)	0.091
Not married or engaged	1.20 (0.89–1.63)	0.235	1.56 (1.05–2.31)	0.027
Widow	0.46 (0.27–0.80)	0.000	0.83 (0.41–1.68)	0.607
**Location**				
Rural	1 (ref.)		1 (ref.)	
Urban	0.85 (0.67–1.07)	0.172	0.62 (0.46–0.83)	0.001
**Education**				
Formal education	1 (ref.)		1 (ref.)	
Informal or no education	2.51 (2.08–3.02)	0.000	1.09 (0.85–1.39)	0.506
**Financial difficulties**				
Yes	1 (ref.)		1 (ref.)	
No	2.61 (2.01–3.39)	0.000	2.31 (1.69–3.15)	0.000
**Age**				
< 34	1 (ref.)		1 (ref.)	
35–54 years	0.65 (0.53–0.79)	0.000	0.97 (0.76–1.25)	0.836
> 55 years	0.87 (0.63–1.21)	0.416	0.91 (0.61.1.37)	0.662

## Discussion

This cross-sectional study explored perceived changes in access to care in Afghanistan after the change of government in August 2021. The majority of participants reported that the sense of security when visiting health facilities and their ability to seek care have improved, while their ability to reach health facilities has remained the same, and their ability to pay for care has worsened. Overall, accessing care in the Anabah catchment area and not being married or engaged resulted in a lower likelihood of having perceived an improvement in access to care after August 2021.

Provision of and access to care in conflict settings is a big challenge for health actors and the general population [16]. The decrease in fighting after the Taliban takeover marked the end of decades of instability in Afghanistan [17], and has reasonably contributed to a perception of improvement in the sense of security and the accessibility of health services in the catchment areas of Kabul and Lashkar-Gah, in the Kabul and Helmand provinces. The decrees issued by the de-facto authorities after August 2021, however, risk jeopardising the sense of security in the country. In particular, those restricting women’s right to education, employment and leisure activities, and those regulating behaviour and appearance in public spaces [18], may have prevented women from perceiving an improvement in the sense of security on par with men. Interestingly, an improvement in the sense of security was more likely to be reported by unmarried/unengaged individuals, possibly because of a greater degree of independence and fewer concerns they have compared to individuals with families. These findings are intriguing and could be usefully explored further in qualitative studies.

The majority of patients perceive an improvement in the ability to seek care after August 2021. In particular, individuals seeking care in the catchment areas of Kabul and Lashkar-Gah were more likely to perceive an improvement than those in Anabah. One explanation may be that areas that predominantly invested in emergency care due to a large influx of war casualties have now redirected their resources to primary and routine care, making services overall more accessible to the population. Nevertheless, traumatic events in Afghanistan are not only attributable to conflict or violence, but they frequently also occur in the context of road traffic accidents [19] or disasters [20]. Overall, lessening the burden of acute ailments on the health system may enable allocation of resources to continuous primary care, possibly contributing to improvements in people’s ability to seek care.

Most patients reported that their ability to reach care did not change after August 2021, suggesting that geographical accessibility is an issue regardless of the change of government and the decrease in fighting. Reasons may include the fact that roads are damaged by mines and explosions, rural and hard-to-reach communities remain uncovered by the health system, public ambulances are insufficient to meet the needs of the population, and the economic crisis prevents families from buying fuel or paying for transports [21–23]. Over the years, initiatives deploying mobile health teams have been set up and proven useful to deliver basic care [24]. However, these initiatives have never been considered the reference point of care for many Afghans and they have recently been compromised by bans on women’s work for international NGOs, which forced several clinics to close [25], pushing remote communities further into marginalisation. Health system reforms should consider the current size and distribution of the population, for all communities to be located at a reasonable distance from a facility. An effective referral system that relies on ambulances and the necessary equipment is a priority to improve reachability of care.

The ability to pay for care has deteriorated for the majority of Afghans after August 2021. Economic hardship and increased costs have undermined the affordability of medical care. Although healthcare is ostensibly free in public hospitals, patients are often required to pay for medicines out-of-pocket [21]. A 2020 study on access to cardiovascular medicines in Afghanistan shows that the public health sector accounts for only 50% of the needed medicines, which means these are still purchased in private pharmacies. This contributes to aggravating the financial situation of families and puts them at risk of catastrophic health expenditure [26]. Households have started adopting short-term coping strategies to survive the economic crisis, including delaying or giving up treatment, borrowing money, accessing humanitarian aid, selling assets or livestock, and child labour. Prioritising food over other goods and services, including healthcare, is another coping mechanism [27].

The results resonate with some common characteristics scholars have pointed out for other post-conflict settings, including the ample exodus of healthcare workers [28], the damaged and/or suboptimal infrastructures and fragmented health service delivery [29], emergency care subject to disruptions in transportation because damaged roads hamper dispatch of ambulances [29], poor coordination and multiplicity of health actors with blurred boundaries between humanitarian relief and health development interventions [30]. Access to care is particularly challenging in these contexts, yet it is a prerequisite for UHC. Post-conflict settings provide an opportunity to develop effective evidence-based interventions and policies to rebuild health systems [28]. Investing in primary care by strengthening the role of community health workers could lead to improved access to care, as they may leverage their physical proximity with community members [29]. This may ultimately contribute to state legitimacy [30].

When it comes to Afghanistan specifically, it has been recognised that this moment could be historically right for reforming the healthcare system [14]. Decisions on services to be included in healthcare packages have to rely on priority setting processes that ensure that packages are equitable and fair, reflect the needs of the population, and are feasible considering the resources available [31]. Ultimately, no health system reform can occur in Afghanistan without a whole-of-society approach, which involves engagement of communities and civil society representatives, availability of competent and fairly distributed human resources, institutionalised managerial and technical capacities, political commitment and country ownership, as well as sustainable healthcare financing [32].

### Limitations and strengths

Our sample was limited to individuals who have visited an EMERGENCY facility at some point during the study period, thus neglecting the views of individuals accessing other types of healthcare services as well as those with no access to care at all. For this reason, it is difficult to assess how much these results apply to the general population in Afghanistan. To partially reduce this potential bias, participants were asked about barriers to access to care in general, not only those faced when accessing EMERGENCY facilities. In this regard, up to 60% of participants reported that they had frequently sought care at government facilities in the past year, which means the identified barriers reasonably extend beyond EMERGENCY services. On a positive note, this study reached respondents from very remote areas, scarcely accessible over the past 20 years. To achieve as much variety as possible, data was collected in provinces with different historical, social, economic, and geographical profiles, and in both urban and rural areas. Of note is the reliance on an established theoretical framework for the conceptualisation and development of the questionnaire, which increases the comparability of results with those of other studies having a similar theoretical approach.

## Conclusions

This study provided for the first time in-depth information on the perceived changes in access to care after the change of government in Afghanistan. The majority of participants perceive that their sense of security and ability to seek care have improved, while their ability to reach care has remained the same, and their ability to pay for care has worsened. Interesting results such as the higher likelihood of unmarried/unengaged individuals to perceive an increased sense of security call for more in-depth qualitative research. This study’s results indicate which dimensions of access to care need resource allocation to be improved and which groups have experienced worse consequences after the government has changed. By highlighting geographical and socio-demographic inequalities in access to care, the findings of this study will ultimately inform the development and implementation of ad hoc interventions aimed at improving access to care.

### Electronic supplementary material

Below is the link to the electronic supplementary material.

Supplementary Material 1

## Data Availability

No datasets were generated or analysed during the current study.

## Footnotes

1 EMERGENCY is also present in a very remote area in the Badakhshan province, but it was not possible for the research team to reach the facility, hence the exclusion of the province from the study.

## Abbreviations

NGO: Non Governmental Organisation
UHC: Universal Health Coverage
US: United States
NATO: North Atlantic Treaty Organisation
FAP: First Aid Post
PHC: Primary Healthcare Centre
OR: Odds Ratio
CI: Confidence Interval
