# Supplementary material for: Access to care in Afghanistan after august 2021: a cross-sectional study exploring Afghans’ perspectives in 10 provinces
Source: Confl Health. 2024 Apr 22;18:34. doi: 10.1186/s13031-024-00594-5 (PMC11034132; doi:10.1186/s13031-024-00594-5)
Supplement: Supplementary file 1 — Supplementary Material 1 [file 13031_2024_594_MOESM1_ESM.pdf]

Supplementary Material

Table of contents

Supplementary material 1: Questionnaire ……….. 1

Supplementary Material 2: Table on perceived changes in different domains of access to care after August 2021……………………………………………….....…… 13

Supplementary Material 1: Questionnaire

TITLE
A Mixed-Methods Study Exploring Access to Healthcare in Different Provinces in Afghanistan

OBJECTIVE
To examine access to healthcare trends and barriers in Afghanistan over the past 3 years, and how these trends and barriers have changed today compared to three years ago.

INCLUSION AND EXCLUSION CRITERIA FOR PARTICIPANTS
Inclusion criteria:
- being an adult (> 18 years old)
- having lived in Afghanistan for the past 3 years or more
- visiting an EMERGENCY facility/being in EMERGENCY premises
- speaking Dari, Pashto, or English
- providing informed consent

Exclusion criteria:
- those who do not meet the above-mentioned inclusion criteria

ETHICAL IMPLICATIONS AND INFORMED CONSENT

Duration
The total duration for completing the questionnaire is 20 minutes.

Risks
There are no direct risks to you as a participant.

Benefits
Although there are no direct benefits to you as a participant, your opinion is highly valued to prompt policy changes and improve health service delivery in Afghanistan.

Reimbursements
There is no reimbursement for this study.

Confidentiality
The study investigators and analysts will take all reasonable precautions to ensure adequate confidentiality of information where possible. Results of the study will be published in aggregate, and your specific responses will not be identified in the published material.

☐ I understand these terms and I consent to take part in this study.

No.	Questions	Coding categories	Skip to
		Pre-filled information	
a)	Name of interviewer	___________	
b)	Date of interview (dd-mm-yyyy)		
c)	Time of interview	___________	
d)	Type of EMERGENCY facility	1. First Aid Post<br>2. First Aid Post/Primary Care Center<br>3. Primary Care Center<br>4. Hospital<br>5. Other: ___________	
e)	Village/city name	____________	
f)	District name	____________	
g)	Province name	____________	
		[Demographic information]	
1	Interviewee's gender	1. Male<br>2. Female	
2	What is your age?	1. Number: _____<br>2. I don't know	
3	What is your marital or engagement status?	1. Married<br>2. Divorced, separated<br>3. Widowed<br>4. Never married, but engaged<br>5. Never married, not engaged	
4	Are you the head of the household?	1. Yes<br>2. No	If answer is 1 → skip to 6<br>If answer is 2 → continue with 5
5	What is your relationship with the head of the household?	______________________ (e.g., husband, wife, son, daughter…)	
6	How many people are there in your household?	Number: _____________	
7	How many people in your household have:<br><br>("*Can you tell me what the approximate age of every member of your family is?*")	5 years or less (number): __________<br>From 6 to 17 years (number): ___________<br>From 18 to 35 years (number): ___________<br>From 36 to 55 years (number): ___________	

		More than 56 years (number): _________	
8	In what village/city do you live now?	Name of village/city: ________________	
9	In what district is that village/city located?	Name of district: ___________________	
10	In what province is that district located?	Name of province: __________________	
11	Do you live in:	1. Urban area (city or suburb, medium-large town)<br>2. Rural area (open countryside, village/small town)<br>3. I don't know	
12	What is the highest educational level in your household?	1. No education<br>2. Primary<br>3. Lower Secondary<br>4. Higher Secondary<br>5. Undergraduate<br>6. Graduate<br>7. Religious/informal education<br>8. I don't know	
13	What is your employment status?	1. Day laborer<br>2. Salaried worker, private sector<br>3. Salaried worker, public sector<br>4. Subsistence farmer<br>5. Self-employed<br>6. Employer<br>7. Unpaid family worker	
[Access to care: general]			
14	What is the main reason why you are seeking care at this EMERGENCY facility today?	1. Medical examination<br>2. Drug prescription<br>3. Health system navigation<br>4. Urgent care<br>5. Accompanying someone<br>6. Need for help in general<br>7. Other: ____________	
15	In the past year, what health facility have you visited most often?	1. Health post<br>2. Health sub-center<br>3. Basic health center<br>4. Mobile health team<br>5. Comprehensive health center<br>6. District hospital<br>7. Provincial/national hospital	

	(*If they mention the name or location of the facility they visited, type it into "other" and we classify later*)<br><br>* more than 1 answer is possible	8. Maternity home<br>9. Visit by health provider at home<br>10. Traditional healers<br>11. I don't know<br>12. Other: __________________	
16	What type of facility was it?<br><br>* more than 1 answer is possible	1. EMERGENCY facility<br>2. Government facility<br>3. National non-governmental organization facility<br>4. International non-governmental organization facility<br>5. Traditional medicine, informal care<br>6. I don't know<br>7. Other: ___________________	
[Approachability of health information]			
17	What is your main source of information about health and health services?<br><br>* more than 1 answer is possible	1. Healthcare providers<br>2. Family and/or friends<br>3. Mass media (television, radio, etc.)<br>4. Internet<br>5. Pamphlets, books<br>6. Support organizations (e.g., third sector organization, non-governmental organizations)<br>7. Community leaders<br>8. Traditional healers<br>9. None<br>10. Other: _____________	
18	Do you consider information about health and health services easy to find?	1. Yes<br>2. No<br>3. I don't know	
19	Do you consider information about health and health services easy to understand?	1. Yes<br>2. No<br>3. I don't know	
20	Has the way you get information about health and health services changed today, compared to 3 years ago?<br><br>("*Overall, would you say it has improved, worsened?*")	1. Improved<br>2. Worsened<br>3. Stayed the same<br>4. I don't know<br>5. Some things have improved, some things have worsened	If answer is 1 → continue with 21<br>If answer is 2 → skip to 22<br>If answer is 3 or 4 → skip to 23<br>If answer is 5, read both 21 & 22

21	For what reasons do you think the situation has improved?	1. More outreach activities<br>2. More security<br>3. More access to media and the internet<br>4. Less difficulty in reaching facilities/more availability of means of transport<br>5. Increased trust in health messages<br>6. Other: ___________________	Any answer → skip to 23
22	For what reasons do you think the situation has worsened?	1. Less outreach activities<br>2. Less security<br>3. Less access to media and the internet<br>4. Transports more difficult<br>5. Diminished trust in health messages<br>6. Less access to money<br>7. Other: ___________________	
[Ability to perceive]			
23	In the past year, did you spend less money on food or clothing to be able to cover healthcare costs (like seeing a doctor, drugs, buying COVID-19 equipment)?	1. Yes<br>2. No<br>3. I don't know	
24	When do you go and see a doctor?<br><br>* more than 1 answer is possible	1. If I have pain or I don't feel well___<br>2. If I have enough money ___<br>3. If someone (friends/family) comes with me ___<br>4. If I find a means of transportation __<br>5. If I have someone who takes care of my kids/family members ___<br>6. I need people to convince me ___<br>7. I need people to give me permission to go<br>8. Other: ____________________	
25	Considering the Afghan health system in general, how satisfied are you with the health services that are available when you fall sick or get injured?	1. Very satisfied<br>2. Satisfied<br>3. Neither satisfied nor unsatisfied<br>4. Unsatisfied<br>5. Very unsatisfied	
26	Has your satisfaction with the health services changed today, compared to 3 years ago?	1. Improved<br>2. Worsened<br>3. Stayed the same<br>4. I don't know<br>5. Some things have improved, some things have worsened	If answer is 1 → continue with 27<br>If answer is 2 → skip to 28<br>If answer is 3 or 4 → skip to 29<br>If answer is 5, read both 27 & 28
27	For what reasons do you think the situation has improved?	1. Better attitude of health professionals<br>2. Better team composition	

		3. Increased availability of services<br>4. Better financial ability<br>5. Healthcare costs more affordable<br>6. More availability of drugs/medicines<br>7. Other: ___________________	Any answer → skip to 29
28	For what reasons do you think the situation has worsened?	1. Worse attitude of health professionals<br>2. Worse team composition<br>3. Diminished availability of services<br>4. Worse financial ability<br>5. Healthcare costs less affordable<br>6. Less availability of drugs/medicines<br>7. Other: ___________________	
[Acceptability]			
29	What is your preference in terms of gender for the facility staff?	1. Male<br>2. Female<br>3. Gender does not matter	
30	Are you generally happy with the gender composition of the staff in health facilities?	1. Yes<br>2. No<br>3. I don't know	
31	In the past year, have you, or any family members or close friends, felt offended or stigmatized when seeking or receiving care at a health facility?	1. Yes<br>2. No<br>3. I don't know	If answer is 1 → continue with 32<br>If answer is 2 → skip to 33<br>If answer is 3 → skip to 33
32	What are the main reasons why you, or any family members or close friends, felt offended or stigmatized when seeking or receiving care at a health facility?<br><br>*(For what characteristics?)*<br><br>* more than 1 answer is possible	1. Ethnicity<br>2. Disability<br>3. Mental health disorders<br>4. Gender<br>5. Income/social status<br>6. Religion<br>7. Educational level<br>8. Other: _________________	
33	Considering the Afghan health system in general, how safe do you feel today when approaching health services?	1. Very safe<br>2. Safe<br>3. Neither safe nor unsafe<br>4. Unsafe<br>5. Very unsafe	

34	Has your sense of safety/security towards the health services changed today, compared to 3 years ago?	1. Improved<br>2. Worsened<br>3. Stayed the same<br>4. I don't know<br>5. Some things have improved, some things have worsened	If answer is 1 → continue with 35<br>If answer is 2 → skip to 36<br>If answer is 3 or 4 → skip to 37<br>If answer is 5, read both 35 & 36
35	For what reasons do you think the situation has improved?	1. Better staff composition<br>2. Less stigmatization<br>3. Staff more welcoming<br>4. Health facilities more secure<br>5. Other: ______________	Any answer → skip to 37
36	For what reasons do you think the situation has worsened?	1. Worse staff composition<br>2. More stigmatization<br>3. Staff less welcoming<br>4. Health facilities more dangerous<br>5. COVID-19<br>6. Other: ______________	
[Ability to seek care]			
37	In the past year, have you, or some of your family members or close friends, encountered any obstacles in accessing care?	1. Yes<br>2. No	If answer is 2 → skip to 39
38	What type of obstacles did you, or your family members or close friends, encounter?<br><br>* more than 1 answer is possible	1. It was too dangerous because of the conflict<br>2. It was too dangerous because I could get mugged<br>3. I could not afford to go to see a doctor<br>4. Health facilities were too far from me<br>5. Health facilities are not designed for people with disability<br>6. It was dangerous because I felt discriminated<br>7. COVID-19<br>8. Insecurity due to disasters (earthquake, floods)<br>9. Other: _______________	
39	In the past year, did you, or some of your family members or close friends, have any concerns that kept you away from seeking care?	1. Yes<br>2. No	If answer is 2 → skip to 41
40	What were these main concerns?	1. I was scared of conflict and violence<br>2. I was scared I had no money<br>3. I had no trust in modern medicine<br>4. I was scared I could be arrested<br>5. I had more pressing issues at home	

		6. I was scared of catching COVID-19<br>7. I was scared because of disasters (earthquake, floods)<br>8. Other: ________________	
41	Did anyone in your family or close friends die because they could not receive care in the past year?	1. Yes<br>2. No<br>3. I don't know	
42	Has your ability to seek care changed today, compared to 3 years ago?	1. Improved<br>2. Worsened<br>3. Stayed the same<br>4. I don't know<br>5. Some things have improved, some things have worsened	If answer is 1 → continue with 43<br>If answer is 2 → skip to 44<br>If answer is 3 and 4 → skip to 45<br>If answer is 5, read both 43 & 44
43	For what reasons do you think the situation has improved?<br><br>* more than 1 answer is possible	1. More facilities<br>2. More healthcare workers<br>3. Easier transportations<br>4. Quality of services has improved<br>5. Improved security<br>6. Reduction in costs<br>7. More drugs available<br>8. Other: ________________	Any answer → skip to 45
44	For what reasons do you think the situation has worsened?<br><br>* more than 1 answer is possible	1. Fewer facilities<br>2. Fewer healthcare workers<br>3. Less transportations<br>4. Quality of services has worsened<br>5. More insecurity and fear<br>6. Higher costs<br>7. Fewer drugs available<br>8. Other: ___________________	
[Ability to reach care/Availability]			
45	In the past year, did you, or any family members or close friends, face any of the following issues when seeking care for your condition(s)?<br><br>* more than 1 answer is possible	1. Go to another city to get the needed service<br>2. Go to another province to get the needed service<br>3. Go to another country to get the needed service<br>4. None of the above	
46	In the past year, have you, or some of your family members or close friends, sought care in the health facility closest to your home?	1. Yes<br>2. No<br>3. I don't know	If answer is 1 → skip to 48<br>If answer 2 → proceed with 47<br>If answer is 3 → skip to 48

47	What are the reasons why you, or some of your family members or close friends, did not visit the health facility closest to your home?<br><br>* more than 1 answer is possible	1. More expensive<br>2. Unfriendly staff behavior<br>3. No trust in ability of health staff<br>4. No trust in quality of services<br>5. Security issues<br>6. Facility was closed<br>7. Facility converted to COVID-19 care only<br>8. Too many COVID-19 cases<br>9. Other: __________________	
48	In the past year, which means of transportation have you, or some of your family members or close friends, used most often to reach the facility?<br><br>* more than 1 answer is possible	1. Walk<br>2. Bike<br>3. Tuk tuk<br>4. Private car or similar vehicle<br>5. Private bus or ambulance<br>6. Ambulance (public)<br>7. Animal (e.g., donkey, horse, mule)<br>8. Other: ______________	
49	In the past year, what has been the average travel time to a health facility for you, or any family members?<br><br>(*If the respondent tells an approximate time in hours or if he/she reports of a distance in km, write that in "other" and we will code later*)	1. Time in minutes: _____________<br>2. I don't know	
50	Overall, how do you consider traveling from your home to the health facility to receive care?	1. Very challenging<br>2. Challenging<br>3. Normal<br>4. Easy<br>5. Very easy	
51	Has your ability to reach care changed today, compared to 3 years ago?	1. Improved<br>2. Worsened<br>3. Stayed the same<br>4. I don't know<br>5. Some things have improved, some things have worsened	If answer is 1 → continue with 52<br>If answer is 2 → skip to 53<br>If answer is 3 and 4 → skip to 54<br>If answer is 5, read both 52 & 53
52	For what reasons do you think the situation has improved?	1. Less fear, insecurity, and safety concerns<br>2. Less costs<br>3. Better opening hours<br>4. Better appointment mechanisms<br>5. More facilities available	Any answer → skip to 54

	* more than 1 answer is possible	6. Better transportation<br>7. Better ambulances/emergency services<br>8. Better mobility<br>9. Other: ________________	
53	For what reasons do you think the situation has worsened?<br><br>* more than 1 answer is possible	1. More security and safety concerns<br>2. More costs<br>3. Worse opening hours<br>4. Worse appointment mechanisms<br>5. Less facilities available<br>6. Worse transportation<br>7. Worse ambulances/emergency services<br>8. Mobility restrictions<br>9. Other: ________________	
[Ability to pay for care]			
54	Have you, or any family members or close friends, ever experienced financial difficulties as a result of spending on healthcare?	1. Yes<br>2. No<br>3. I don't know	
55	Considering care sought by you, or any of your family members or close friends in the past year, what do you think about the costs?	1. Very expensive<br>2. Expensive<br>3. Normal<br>4. Inexpensive<br>5. Very inexpensive<br>6. I don't know	
56	What did you spend the most money on?	1. Medications<br>2. Treatment/care<br>3. Travel/transportation to healthcare facilities<br>4. I don't know<br>5. Other: _____________	
57	In the past year, did you, or any family members or close friends, forego or postpone healthcare visits because of cost?	1. Yes<br>2. No<br>3. I don't know	
58	Have you, or any family members or close friends, ever been forced to borrow money or sell goods to obtain healthcare in the past year?	1. Yes<br>2. No<br>3. I don't know	
59	Has your ability to pay for care changed today, compared to 3 years ago?	1. Improved<br>2. Worsened<br>3. Stayed the same	If answer is 1 → continue with 60<br>If answer is 2 → skip to 61<br>If answer is 3 or 4 → skip to 62

		4. I don't know<br>5. Some things have improved, some things have worsened	If answer is 5, read both 60 & 61
60	For what reasons do you think the situation has improved?	1. Income has improved; availability of more money<br>2. Treatment costs are lower<br>3. Transportation is cheaper<br>4. Other: _______________	Any answer → Skip to 62
61	For what reasons do you think the situation has worsened?	1. Income has decreased; availability of less money<br>2. Treatment costs are higher<br>3. Transportation is more expensive<br>4. Other: _______________	
[Appropriateness]			
62	Considering care sought by you, or by any of your family members or close friends in the past year, have you experienced any significant delay in accessing:<br><br>* more than 1 answer is possible	1. Drugs/Medicines ___<br>2. Surgery or other procedures ___<br>3. Diagnostic tests ___<br>4. Appointment with primary care doctor ___<br>5. Appointment with a specialist doctor ___<br>6. Other: ____________________	
63	Considering care sought by you, or by any of your family members or close friends in the past year, what do you think of the staff behavior?	1. Very good<br>2. Good<br>3. Normal<br>4. Bad<br>5. Very bad	If answer 1,2, 3 → proceed to 65<br>If answer 4 or 5 → proceed to 64
64	What were the reasons for this poor staff behavior?	1. Violent and aggressive behavior<br>2. Verbal abuse<br>3. Neglectfulness<br>4. Incompetence<br>5. Corruption<br>6. Other: _____________	
65	In the past year, have you, or any family members or close friends, always gotten access to the drugs/medicines you needed?	1. Yes<br>2. No<br>3. I don't know<br>4. Never needed any drugs	
66	Overall, has access to care changed today, compared to 3 years ago?	1. Improved<br>2. Worsened<br>3. Stayed the same<br>4. I don't know	

67	Overall, how do you think access to healthcare can be improved?<br><br>* more than 1 answer is possible	1. Reduction of travel time<br>2. Reduction of travel costs<br>3. Reduction of care costs<br>4. Better staff attitude<br>5. Improvement of quality of services<br>6. Deployment of more female staff<br>7. Increased number of health facilities<br>8. Other: ____________________	

Supplementary Material 2: Table on perceived changes in different domains of access to care after August 2021

Perceived changes in access to care after August 2021, disaggregated by gender and project.

**Perceived changes in access to care**	Gender N(%)		Project N(%)				Total N(%)
	Female	Male	Anabah	Kabul	Lashkar Gah	Unknown	
**Change in the sense of security**							
Improved	114 (22.27)	862 (66.56)	121 (17.11)	307 (61.03)	547 (92.24)	1 (25.0)	976 (54.34)
Neither improved nor worsened	279 (54.49)	345 (26.64)	413 (58.42)	165 (32.80)	43 (7.25)	3 (75.0)	624 (34.74)
Worsened	116 (22.66)	80 (6.18)	168 (23.76)	26 (5.17)	2 (0.34)	NA	196 (10.91)
**Change in the ability to seek care**							
Improved	73 (14.26)	567 (43.78)	61 (8.63)	107 (21.27)	472 (79.60)	NA	640 (35.63)
Neither improved nor worsened	184 (35.94)	368 (28.42)	253 (35.79)	205 (40.76)	92 (15.51)	2 (50.0)	552 (30.73)
Worsened	252 (49.22)	352 (27.18)	388 (54.88)	186 (36.98)	28 (4.72)	2 (50.0)	604 (33.63)
**Change in the ability to reach health facilities**							
Improved	73 (14.26)	588 (45.41)	64 (9.05)	143 (28.43)	454 (76.56)	NA	661 (36.80)
Neither improved nor worsened	336 (65.63)	567 (43.78)	495 (70.01)	288 (57.26)	119 (20.07)	1 (25.0)	903 (50.28)
Worsened	100 (19.53)	132 (10.19)	143 (20.23)	67 (13.32)	19 (3.20)	3 (75.0)	232 (12.92)
**Change in the ability to pay for healthcare**							
Improved	32 (6.25)	388 (29.96)	31 (4.38)	40 (7.95)	349 (58.85)	NA	420 (23.39)
Neither improved nor worsened	147 (28.71)	406 (31.35)	149 (21.07)	206 (40.95)	196 (33.05)	2 (50.0)	553 (30.79)
Worsened	330 (64.45)	493 (38.07)	522 (73.83)	252 (49.90)	47 (7.93)	2 (50.0)	823 (45.82)
